# First Report on Smoking and Infection Control Behaviours at Outdoor Hotspots during the COVID-19 Pandemic: An Unobtrusive Observational Study

**DOI:** 10.3390/ijerph18031031

**Published:** 2021-01-25

**Authors:** Yuying Sun, Tai Hing Lam, Yee Tak Derek Cheung, Man Ping Wang, Yongda Wu, Jianjiu Chen, Xiaoyu Zhang, William H. C. Li, Sai Yin Ho

**Affiliations:** 1 School of Public Health, The University of Hong Kong, Hong Kong, China; gyysun@hku.hk (Y.S.); hrmrlth@hku.hk (T.H.L.); jjchen@connect.hku.hk (J.C.); xiaoyu66@connect.hku.hk (X.Z.); syho@hku.hk (S.Y.H.); 2 School of Nursing, The University of Hong Kong, Hong Kong, China; mpwang@hku.hk (M.P.W.); yongdang@connect.hku.hk (Y.W.); william3@hku.hk (W.H.C.L.)

**Keywords:** smoker, smoking, COVID-19, infection control behaviours, face mask

## Abstract

This study was to observe smoking behaviours and infection control behaviours in smokers at outdoor smoking hotspots during the COVID-19 pandemic in Hong Kong. We conducted unobtrusive observations at nine hotspots during 1 July 2019–31 January 2020 (pre-outbreak, 39 observations), 1 February–30 April 2020 (outbreak, eight observations), and 1 May–11 June 2020 (since-outbreak, 20 observations). Sex, age group, type of tobacco products used, duration of stay, group smoking behaviours, face mask wearing and infection control behaviours of smokers, and mask wearing of non-smoking pedestrians were observed. Compared with pre-outbreak, lower volumes of smokers were observed during outbreak and since-outbreak. Smokers gathered more in a group (24.5% and 25.8% vs. 13.4%, respectively) and stayed longer (91.5% and 83.6% vs. 80.6% stayed ≥1 min) during outbreak and since-outbreak than pre-outbreak. Ninety-six percent smokers possessed a face mask. While smoking, 81.6% of smokers put the mask under the chin and 13.8% carried it in the hand, 32.4% did not wear a mask immediately after smoking, 98.0% did not sanitize hands, and 74.3% did not keep a distance of at least one metre. During the COVID-19 pandemic, smokers gathered closely and stayed longer at the hotspots, and few practised hand hygiene, all of which may increase the risk of infection.

## 1. Introduction

Smoking is likely associated with more severe outcomes of COVID-19 [1,2], but the controversy over whether smoking will increase the infection risk of COVID-19 is ongoing [3,4,5]. Smoking, with repetitive hand and mouth contacts, may put smokers at higher risk of infection, since the predominant transmission routes of COVID-19 are respiratory droplets, personal contact, and contaminated surfaces [6,7]. Although being the most developed and wealthiest city of China and among countries and regions with the lowest smoking prevalence, Hong Kong still has a large number of daily smokers (652,000; 10.5% of all persons aged over 15 years) [8]. Outdoor smoking hotspots have become the most common public places where smokers gather to smoke since the comprehensive indoor smoke-free legislation has taken effect on 1 January 2007 [9]. We have previously described such hotspots and our success in recruiting smokers for smoking cessation projects [10,11,12]. Smoking hotspots were selected if (1) group smokers were observed; (2) a rubbish bin with an ashtray for collecting cigarette butts was nearby; and (3) the observation area had a large number of pedestrians, including smokers and non-smokers [12]. They are open spaces near exits of mass transit railway stations, and entrances of shopping plazas and transport hubs, typically with a rubbish bin for collecting cigarette butts. Group smoking typically involves two or more smokers, standing closely or talking face-to-face in a circle. Some studies elsewhere suggested that group smoking at outdoor public areas restored the smokers’ social identity and increased their solidarity [13,14]. Talking and smoking at hotspots may help smokers fulfil their social needs of maintaining social connection. However, respiratory droplets produced while talking or exhaling smoke may endanger others if the smoker is infected by COVID-19. Therefore, hotspot smoking is potentially a high-risk behaviour for transmitting COVID-19. In July 2020 in Hong Kong, a COVID-19 case was reported to have close contacts with smokers at a smoking hotspot [15]. Our search of PubMed on 12 January 2021 using keywords of “COVID-19” or “SARS-CoV-2”, or “coronavirus” and “smoking”, showed no reports on smoking at hotspots and the infection control measures taken by smokers.

The first confirmed case of COVID-19 was reported in Hong Kong on 23 January 2020, the same day lockdown began in Wuhan, China. The first wave in Hong Kong reached the peak of 10 daily cases on 9 February. Special work arrangement for government departments began on 29 January [16], allowing work from home except for emergency and essential services. Many private sector organisations followed suit. The second wave reached the peak of 65 daily cases on 27 March. In response, the government closed 11 types of businesses since 28 March and banned public gatherings including dining of more than four people since 29 March. With five days of zero local cases during 20 to 28 April, the government relaxed the maximum gathering group size to eight from 8 May (effective until 18 June) [17].

The special work arrangements and social distancing measures have markedly reduced the volume of pedestrians in outdoor areas. However, the effects on the volume of smokers at hotspots and their smoking related behaviours were unclear. The World Health Organization (WHO) recommends 1 metre (3 feet) of social distance [18] and the US Centers for Disease Control (CDC) suggests 2 metres (6 feet) to avoid person-to-person spread of COVID-19 [19]. The Hong Kong government recommends a distance of at least one metre from others, but whether this was followed by smokers at smoking hotspots is unknown.

In Hong Kong, mass masking had been advocated early before COVID-19 was declared a pandemic as a useful adjunct to social distancing and hand hygiene [20]. Face mask wearing by the general public in Hong Kong was 97.5% in February and 98.8% in March from a telephone survey, and was 96.6% in April 2020 from an outdoor observational study [21,22]. The prevalence of mask wearing in smokers and how they handle the mask during smoking are unknown. With no convenient hand washing facilities near the smoking hotspots, whether smokers use hand sanitizer before and after smoking is also unknown.

This first unobtrusive observational study aimed to investigate: (1) the changes in volumes (persons per hour) of smokers from 1 July 2019–31 January 2020 (pre-outbreak period), to 1 February–30 April 2020 (outbreak period, i.e., 1st and 2nd wave), and 1 May–11 June 2020 (since-outbreak period, i.e., post 2nd wave), and whether similar changes occurred for pedestrians in general at the hotspots (including those who were not smoking), (2) smokers’ group smoking behaviours during the above three periods, and (3) smokers’ infection control behaviours from 20 May to 11 June 2020 (in the since-outbreak period). A total of 67 observations were conducted at noon (11 am to 2 pm) and in the afternoon (3 to 6 pm) on weekdays from 1 July 2019 and 11 June 2020. We started doing hotspot observations from July 2019 as part of a project on tobacco control policy commissioned by the government. Since the COVID-19 outbreak, the observations included COVID-19 related behaviours, although they were interrupted to protect our observers when case numbers were high.

## 2. Materials and Methods

### 2.1. Location of Observations

An observational study was conducted at smoking hotspots to measure the volumes (persons per hour) of smoking and non-smoking pedestrians, and to observe their behaviours. Ethical approval was granted by the Institutional Review Board of the University of Hong Kong/Hospital Authority Hong Kong West Cluster (No. UW19-169, date of approval: 23/4/2019, revision approved on 20/5/2020).

Based on our previous studies and pilot observations in different districts [10,11], we selected nine hotspots with the largest number of pedestrians and smokers in Hong Kong Island (four sites: Admiralty, Hong Kong Station, Causeway Bay, and Sheung Wan), Kowloon (three sites: Tsim Sha Tsui, Mong Kok, and Kwun Tong) and the New Territories (two sites: Tsuen Wan and Kwai Fong). The selected sites should be feasible to conduct multiple unobtrusive observations from a distance of at least 10 metres. The boundaries of each hotspot were clearly defined by fixed structures (e.g., poles, walls, and curbs) or markings in the environment to facilitate observations by two observers. The size of each hotspot was around 20 m^2^. People who were holding or using any tobacco products were recorded as smokers, and otherwise as non-smoking pedestrians. Each hotspot was observed 6–10 times, with a total of 39 observations during the pre-outbreak period, eight during outbreak, and 20 since-outbreak. Apart from the district of the hotspot location (Hong Kong Island, Kowloon, and the New Territories), the observation time of the day (noon: 11 a.m.–2 p.m., afternoon: 3 p.m.–6 p.m.), weather (rainy, sunny, or cloudy), and the average temperature of the day were also considered as possible determinants of the volume.

### 2.2. Procedures

A total of 35 observers were trained by the research team. Two observers were needed for each session to record smokers and non-smoking pedestrians unobtrusively. All observations were conducted in noon or afternoon sessions on weekdays when the volume was largest, each lasting for 3 h. During the observations, one observer recorded the smokers, while another observer recorded the non-smoking pedestrians. Smoker’s sex (male, female), age group (adolescents under 21, young adults 21–40, middle-aged 41–60, and elderly >60), types of tobacco products used (conventional cigarettes, e-cigarettes, heated tobacco products [HTPs], and others), duration of stay (passer-by, <1 min, ≥1 min), group smoking behaviours (if two or more smokers appeared to be smoking together as evidenced by staying close, facing each other or chatting), and the number of cigarettes consumed by each smoker (if they used two sticks or more during their stay at the hotspots) were recorded. In the training session, a set of photos of smokers taken on streets were provided to the observers for establishing a consistent standard on judging the age group. The total numbers of non-smoking pedestrians passing by (sex and age group) were recorded concurrently. The 3-h observations were separated into six sessions, with each session lasting for 30 min. Between the sessions, the observers could take short breaks or exchange the roles to avoid fatigue effect, e.g., change from observing smokers to pedestrians. It was common to record large number of pedestrians passing by in a short period. To save the time for each record and to reduce error, the observer only made a simple mark in the corresponding sex/age column (eight columns in total) during the observation and calculated the total number under each category after they had completed all the sessions. A new session always began on a new worksheet to avoid confusion with the previous records.

### 2.3. Observation Forms

The observation forms (smokers: sex, age group, types of tobacco used, and duration of stay; non-smoking pedestrians: sex, age group) were validated by two observers in June 2019. Two pilot 1-h observations of smokers and non-smoking pedestrians on two smoking hotspots (sites AD and UN) were conducted by two observers independently and simultaneously. Interrater reliability of the form was assessed by intra-class correlation coefficient (ICC) and consistency, for smokers and non-smoking pedestrians, respectively. We used ICC to assess the forms of smokers, since the data were individual-based and could be matched between the two observers. Matching was based on specific appearances (e.g., wearing a pink T-shirt, wearing a hat) and confirmed between the two observers immediately. We assessed the consistency of the forms of non-smoking pedestrians, because the data were aggregated numbers in eight sex-age subgroups (two categories of sex, four categories of age).

Whether smokers possessed a face mask and non-smoking pedestrians wore a face mask were added to the form from 4 May to 11 June 2020 during the since-outbreak period. After several sessions of observation, the following infection control behaviours were also documented during 20 May to 11 June 2020: handling of the mask while smoking (put under the chin, carry in the hand, put in the pocket, etc.), wearing a mask immediately after smoking, sanitizing hands at least once during the stay, and maintaining social distance with other smokers (more than one metre).

### 2.4. Statistical Analysis

ICC estimates and their 95% confidence intervals were calculated based on a mean-rating (k = 2), consistency, two-way random effects model [23]. Values of ICC < 0.5, 0.5–0.75, 0.75–0.9, and > 0.9 indicate poor, moderate, good, and excellent reliability, respectively [23]. The consistency was calculated by summing up the consistent numbers of non-smoking pedestrians in the eight sex–age subgroups divided by the average total number of non-smoking pedestrians observed by the two observers (consistency of sex = [consistent number of female + male]/total number of participants; consistency of age = [consistent number of adolescents + young adults + middle-aged + elderly]/total number of participants).

The volumes (hourly counts) of smokers were used as the outcome variable in the multilevel mixed effects model to evaluate the differences in three periods, after adjusting for observation time of the day, district, weather, temperature, and the clustering effect of location (nine sites). The mixed effects Poisson regression model was used to examine the change of the incidence rate of smokers, using the total number of pedestrians (sum of smokers and non-smoking pedestrians) per hour as offset (taking into account all the people of interest) after adjusting for the above factors. Rate ratio refers to the incidence rate ratio (IRR) [24]. An IRR with a *p* < 0.05 for two-tailed test indicates a significant difference in the rate of smokers. We fitted the models using Stata 13 (StataCorp. 2013. Stata Statistical Software: Release 13. College Station, TX, USA: StataCorp LP.). The daily mean temperature (°C) was not recorded during observations, but was obtained from Hong Kong Observatory [25]. The change of volumes of smokers and non-smoking pedestrians across the month was evaluated using a non-parametric test for trend as implemented in Stata [26]. The features of smokers and non-smoking pedestrians at smoking hotspots during the three periods were compared using logistic regression after adjusting for sex, age, type of tobacco products used, duration of stay, group smoking, and number of cigarettes consumed. The infection control behaviours of group smokers and single smokers were compared using Chi-square tests. All figures were generated using R version 4.0.2.

## 3. Results

### 3.1. Validation of the Observation Forms

The smokers (*n* = 166) and non-smoking pedestrians (*n* = 490) were observed by two observers simultaneously. The ICCs of sex (0.98, 95% CI 0.97 to 0.99), age groups (0.79, 95% CI 0.71 to 0.84), type of tobacco products used (0.90, 95% CI 0.86 to 0.93), and duration of stay (0.95, 95% CI 0.93 to 0.96) indicated moderate to excellent reliability of the observations on smokers. The consistency of sex and age group of non-smoking pedestrians were both above 0.90 (0.97 and 0.901, respectively).

### 3.2. Volumes of Smokers

**Figure 1:**
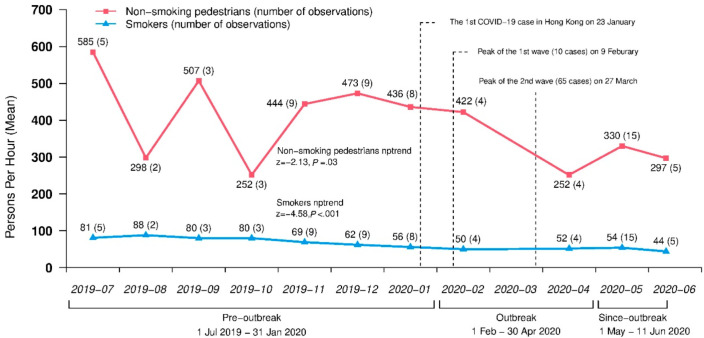
Monthly volumes of smokers and non-smoking pedestrians during July 2019 to June 2020.

A total of 12,107 smokers were observed in 67 observations at nine hotspots during 1 July 2019 to 11 June 2020. Figure 1 shows decreasing trends in the unadjusted monthly volumes of smokers (nptrend z = −4.58, *p* < 0.001) and non-smoking pedestrians (nptrend z = −2.13, *p* = 0.03) (see volumes of smokers at each smoking hotspot in Supplementary Table S1).

Table 1 shows the results from the Poisson regression model. The volumes (persons per hour) of smokers reduced significantly during outbreak and since-outbreak periods (outbreak vs. pre-outbreak: adjusted mean difference, −13.1, 95% CI −24.1 to −2.2; *p* = 0.02; since-outbreak vs. pre-outbreak: adjusted mean difference, −22.0, 95% CI −31.3 to −12.7; *p* < 0.001). The rate of smokers’ since-outbreak was significantly lower than pre-outbreak by 14% (adjusted IRR, 0.86, 95% CI 0.79 to 0.94; *p* = 0.001).

### 3.3. Group Smoking Behaviours

Table 2 shows most smokers were male (72.2%), young adults (56.5%) or middle-aged (36.7%), and used conventional cigarettes (92.8%). Table 2 shows higher proportions of group smoking during outbreak (24.5%) and since-outbreak (25.8%) periods than pre-outbreak (13.4%). The smokers stayed longer in hotspots during outbreak and since-outbreak than pre-outbreak (duration of stay ≥1 min: 91.5% and 83.6% vs. 80.6%).

### 3.4. Infection Control Behaviours since the COVID-19 Outbreak

Table 3 shows that from 4 May to 11 June 2020 during the since-outbreak period, 96.0% smokers possessed a mask while smoking and 98.8% non-smoking pedestrians wore a mask. Online Supplementary Figure S1 shows the day-to-day mask possession of smokers and mask wearing of non-smoking pedestrians during the same period. The proportions of mask wearing of non-smoking pedestrians were all above 97%. The proportion of mask possession in smokers was lower than 85% (84.7% and 83.0%, respectively) on 3 June and 9 June, and was 91.7% on 12 May, although the proportions on the other days were all above 95%.

Table 3 shows 81.6% smokers put their masks under the chin, 13.8% carried in the hand, and only 2.0% sanitized their hands at least once during the stay. One-third (32.4%) did not wear a mask immediately after smoking. About three-quarters (74.3%) were within 1 metre from other smokers while smoking. The proportion of maintaining social distancing (at least 1 metre apart) in group smokers was significantly lower than that in single smokers (7.5% vs. 31.1%, *p* < 0.001). In the group smokers, 325 were with two smokers and 140 were with three or more smokers, of which 91.4% and 95.0% were within one metre from each other, respectively.

## 4. Discussion

This is the first unobtrusive observational study of hotspot smoking during the COVID-19 pandemic, which comprehensively assessed the volume of smokers at these spots and their infection control behaviours during smoking. The proportion of face mask possession in smokers and mask wearing in non-smoking pedestrians was, respectively, 96.0% and 98.8% in May 2020, which is similar to the proportions reported by two earlier Hong Kong studies [21,22]. The almost complete masking was voluntary until the third wave, when the government implemented mandatory masking in outdoor public places from 29 July.

Our study showed that the volume of smokers decreased significantly in both the outbreak and since-outbreak periods. However, the rate of smokers among all pedestrians during outbreak was even higher than pre-outbreak, although the adjusted incidence rate ratio was not significant. This might be due to the insufficient number of observations in the outbreak period, as we cancelled some sessions to protect our observers. Nevertheless, our results showed that smokers’ presence decreased by a smaller extent than the pedestrians at smoking hotspots, which could be explained by smokers’ craving and higher priority to smoke over the need to prevent infection of COVID-19. Group smoking became more common in hotspot smokers during the outbreak and since-outbreak periods, just when public gatherings were limited to four or eight people from 29 March to 18 June. Furthermore, the smokers stayed longer at the hotspots during the outbreak than before. A recent study showed that the COVID-19 pandemic may enlarge the extent and severity of addictive disorders including smoking due to social distancing and poverty, poor mental health, and insecurity that were exacerbated by the pandemic [27]. With usual social activities such as lunch gatherings much affected by the outbreak, some smokers who still visited the hotspots might exploit such opportunities to socialise with other smokers. Our results may be relevant to other high-density urban settings where smoking is prohibited in public indoor places and people must smoke outdoors. Group smoking behaviours are also common in other regions, but may be in different outdoor settings [28]. In Japan, smoking on the street is also allowed if smokers could find an ashtray or remain at one place [29]. A study in the United States has reported multiple smokers gathered at outdoor public places such as parks, sidewalk cafes, restaurants, and pub patios [30].

Apart from the physiological factors that render smokers more susceptible to COVID-19 infection [31], hotspot smoking may also put smokers at a higher risk of infection. We observed that more smokers did not wear masks during the since-outbreak period, while the non-smoking pedestrians were more adherent to mask wearing. Gathering and chatting without masks were common, and most smokers had unprotective behaviours in mask handling, hand hygiene and social distancing. About three quarters of smokers (74.3%) at smoking hotspots did not keep themselves one metre apart. It was unclear whether the smokers had washed their hands before coming out. Even if they had, hand contamination might occur when they used the lift or opened the doors. To reduce the infection risk, public education should emphasize the high risk for hotspot smoking and urge smokers to quit smoking.

Our study had several limitations. First, our observation sessions were suspended for nine weeks due to safety concerns during the peak outbreak period (Mid-February to Mid-April 2020) of COVID-19 in Hong Kong. Second, the observation times and locations during the outbreak and since-outbreak were not fully comparable. This was because we had nine smoking hotspots in different districts but only eight observations covering six sites during the outbreak period. Although we adjusted for several factors, residual confounding could not be ruled out. Third, we tried to cover those hotspots with the largest number of pedestrians to observe more smokers. Therefore, the selected sites might not be representative of all the smoking hotspots, and the characteristics of smokers at hotspots could not represent all smokers in Hong Kong. Lastly, the characteristics of the smokers were different during three periods, thus the infection control behaviours of smokers during since-outbreak period might not represent the smokers observed in pre-outbreak and outbreak periods.

## 5. Conclusions

We first reported that smokers gathered closely and stayed longer while smoking at outdoor smoking hotspots during the COVID-19 pandemic. Removal of face masks and poor hand hygiene were also common high risk behaviours that can increase the risk of infection. Group smoking not only puts the smokers at higher risk, but also may become a transmission route of COVID-19. Besides outdoor smoking hotspots, tobacco use is also common in bars in both Hong Kong and other regions. As the pandemic continues, and evidence of increasing risk of infection and more serious consequences for smokers emerging, smoking cessation campaigns and assistance are urgently needed. Warnings against unmasking for smoking and chatting in groups are needed at smoking hotspots and in mass media campaigns against COVID-19. Smokers should keep at least one metre apart to avoid spreading COVID-19. Due to the increasing severity of the third wave of COVID-19 outbreak in Hong Kong, the government further introduced mandatory masking in outdoor public places and clearly warned that unmasking for smoking was not exempted. Such measures are needed beyond Hong Kong, and could be an opportunity to motivate and support more smokers to quit in Hong Kong and other regions.

## Figures and Tables

**Table 1:** The volumes (persons per hour) of smokers at smoking hotspots in different periods.

Factors	Number of Observations	Unadjusted Mean (SD), Persons Per Hour	Adjusted Mean Difference (95% CI) ^c^	Number of Smokers Per 1000 Pedestrians (SD)	Adjusted Incidence Rate Ratios (95% CI) ^d^
Period ^a^					
Pre-outbreak	39	68.9 (18.7)	Ref.	174.6 (99.0)	Ref.
Outbreak	8	50.8 (18.4)	−13.1 (−24.1, −2.2) *	208.5 (169.7)	1.09 (0.98, 1.23)
Since-outbreak	20	51.6 (15.6)	−22.0 (−31.3, −12.7) ***	166.1 (73.4)	0.86 (0.79, 0.94) **
Observation time of the day ^b^					
Noon	44	58.6 (19.7)	Ref.	176.7 (112.1)	Ref.
Afternoon	23	67.2 (18.6)	6.0 (−4.5, 16.4)	174.9 (91.5)	1.14 (1.01, 1.29) *
District					
Kowloon	23	58.6 (18.0)	Ref.	88.6 (45.6)	Ref.
New Territories	14	67.6 (23.2)	7.4 (−8.2, 23.1)	191.9 (62.2)	2.11 (1.34, 3.32) **
Hong Kong Island	30	61.1 (19.0)	3.7 (−9.3, 16.6)	235.9 (102.3)	2.65 (1.81, 3.87) ***
Weather					
Sunny or cloudy	62	63.5 (18.9)	Ref.	174.7 (103.1)	Ref.
Rainy	5	38.0 (11.0)	−15.0 (−28.4, −1.5) *	193.8 (96.5)	0.96 (0.82, 1.13)
Temperature (°C)	-	-	-	-	1.02 (1.02, 1.03) ***
Total number of pedestrians per hour	67	459.7 (272.4)	-	-	1.0 (offset)

^a^ Pre-outbreak period: 1 July 2019–31 January 2020. Outbreak period: 1 February–30 April 2020. Since-outbreak period: 1 May–11 June 2020. ^b^ Noon: 11 a.m.–2 p.m., Afternoon: 3–6 p.m. ^c^ Adjusted mean difference for observation time of the day, district, weather and temperature (continues variable), and clustering effect of location. ^d^ Adjusted incidence rate ratios for observation time of the day, district, weather and temperature (continuous variable, incidence rate ratios > 1 indicated higher temperature is associated with larger rate of smokers), clustering effect of location, and total number of pedestrians (sum of smokers and non-smoking pedestrians) per hour as offset (taking into account all the people of interest). * *p* < 0.05, ** *p* < 0.01, *** *p* < 0.001.

**Table 2:** Characteristics of smokers and smoking related behaviours at smoking hotspots in three periods.

Factors	Pre-Outbreak Period ^a^ *n* (%) (A)	Outbreak Period ^b^ *n* (%) (B)	Since-Outbreak Period ^c^ *n* (%) (C)	Adjusted Odds Ratio (95% CI) (B) vs. (A)	Adjusted Odds Ratio (95% CI) (C) vs. (A)
Sex					
Male	5664 (72.2)	903 (74.9)	2171 (70.9)	Ref.	Ref.
Female	2176 (27.8)	303 (25.1)	890 (29.1)	0.84 (0.73, 0.97) *	1.02 (0.93, 1.12)
Age ^d^					
Adolescents	73 (0.9)	8 (0.7)	52 (1.7)	Ref.	Ref.
Young adults	4361 (55.6)	672 (55.7)	1801 (59.0)	1.31 (0.62, 2.75)	0.56 (0.39, 0.81) **
Middle-age	2928 (37.3)	442 (36.7)	1072 (35.1)	1.33 (0.63, 2.81)	0.53 (0.37, 0.77) **
Elderly	478 (6.1)	84 (7.0)	130 (4.3)	1.60 (0.74, 3.46)	0.41 (0.27, 0.63) ***
Type of tobacco products used ^e^					
Conventional cigarettes	7217 (92.3)	1147 (95.2)	2853 (93.2)	Ref.	Ref.
E-cigarettes	91 (1.2)	7 (0.6)	75 (2.5)	0.53 (0.24, 1.14)	2.11 (1.54, 2.90) ***
Heated tobacco products	515 (6.6)	51 (4.2)	133 (4.3)	0.62 (0.46, 0.84) **	0.63 (0.52, 0.77) ***
Duration of stay ^f^					
Passer-by	829 (10.6)	62 (5.1)	230 (7.6)	Ref.	Ref.
<1 min	690 (8.8)	40 (3.3)	265 (8.8)	0.76 (0.50, 1.15)	1.36 (1.11, 1.67) **
≥1 min	6311 (80.6)	1104 (91.5)	2526 (83.6)	2.13 (1.63, 2.79) ***	1.25 (1.07, 1.46) **
Single or group smokers					
Single smokers	6789 (86.6)	910 (75.5)	2271 (74.2)	Ref.	Ref.
Two smokers	800 (10.2)	228 (18.9)	541 (17.7)	1.97 (1.67, 2.32) ***	1.92 (1.70, 2.17) ***
Three smokers or more	251 (3.2)	68 (5.6)	249 (8.1)	1.80 (1.36, 2.38) ***	2.91 (2.42, 3.49) ***
Number of cigarettes consumed					
One	7717 (98.4)	1193 (98.9)	2959 (96.7)	Ref.	Ref.
Two or more	123 (1.6)	13 (1.1)	102 (3.3)	0.55 (0.31, 0.98) *	1.95 (1.48, 2.56) ***
Total	7840 (100.0)	1206 (100.0)	3061 (100.0)		

^a^ Pre-outbreak period: 1 July 2019–31 January 2020. ^b^ Outbreak period: 1 February–30 April 2020. ^c^ Since-outbreak period: 1 May–11 June 2020. ^d^ 6 (0.05%) record was missing. ^e^ 18 (0.1%) records were missing. ^f^ 50 (0.4%) records were missing. * *p* < 0.05, ** *p* < 0.01, *** *p* < 0.001.

**Table 3:** Infection control behaviours of smokers and mask wearing of non-smoking pedestrians at smoking hotspots.

Factors	Total *n* (%)	Group Smokers *n* (%)	Single Smokers *n* (%)	Chi-Square	*p*
Smokers					
Mask possession ^a^					
Yes	2936 (96.0)	767 (97.1)	2169 (95.6)	3.40	0.07
No	123 (4.0)	23 (2.9)	100 (4.4)		
Mask handling ^b^					
Put under the chin	1690 (81.6)	415 (81.4)	1275 (81.7)	3.19	0.53
Carry in hand	285 (13.8)	74 (14.5)	211 (13.5)		
Put in pocket	60 (2.9)	14 (2.7)	46 (2.9)		
Hang on ear	30 (1.4)	7 (1.4)	23 (1.5)		
Wearing	5 (0.2)	0 (0)	5 (0.3)		
Wearing masks immediately after smoking ^c^					
Yes	1346 (67.6)	346 (70.3)	1000 (66.7)	2.21	0.14
No	645 (32.4)	146 (29.7)	499 (33.3)		
Sanitizing hands at least once during the stay ^d^					
Yes	42 (2.0)	16 (3.2)	26 (1.7)	4.65	0.03
No	2022 (98.0)	480 (96.8)	1542 (98.3)		
Social distance with others during smoking ^e^					
<1 metre	1516 (74.3)	430 (92.5)	1086 (68.9)	104.35	<0.001
≥1 metre	525 (25.7)	35 (7.5)	490 (31.1)		
Non-smoking pedestrians					
Wearing mask properly	13,790 (95.4)	-	-	-	-
Not wearing mask properly	487 (3.4)	-	-		
No mask at all	182 (1.2)	-	-		

^a^ 2 (0.1%) were missing from 3061 cases recorded from 4 May 2020 to 11 June 2020. ^b^ 11 (0.5%) were missing from 2081 cases recorded from 20 May 2020 to 11 June 2020.^c^ 104 (5.0%) were missing from 2095 cases recorded from 20 May 2020 to 11 June 2020. ^d^ 94 (4.4%) were missing from 2158 cases recorded from 20 May 2020 to 11 June 2020. ^e^ 117 (5.4%) were missing from 2158 cases recorded from 20 May 2020 to 11 June 2020.

## Data Availability

The data presented in this study are available on reasonable request from the corresponding author. The data are not publicly available, because the project is a part of a tobacco control policies study commissioned by the government and the data will only be available upon approval of the funder.

## Supplementary Materials

The following are available online at https://www.mdpi.com/1660-4601/18/3/1031/s1: Figure S1: Mask carrying of smokers and mask wearing of non-smoking pedestrians during since-outbreak period, Table S1: Volumes of smokers and non-smoking pedestrians at 9 smoking hotspots during 1 July 2019 to 11 June 2020.
