# Supplementary material for: First Report on Smoking and Infection Control Behaviours at Outdoor Hotspots during the COVID-19 Pandemic: An Unobtrusive Observational Study
Source: Int J Environ Res Public Health. 2021 Jan 25;18(3):1031. doi: 10.3390/ijerph18031031 (PMC7908604; doi:10.3390/ijerph18031031)

Table S1. Volumes of smokers and non-smoking pedestrians at 9 smoking hotspots during 1 July 2019 to 11 June 2020

	Admiralty	HK station	Causeway Bay	Sheung Wan	Tsim Sha Tsui	Mong Kok	Kwun Tong	Tsuen Wan	Kwai Fong
Smokers, persons per hour									
July 2019	97	-	89	-	84	69	64	-	-
August 2019	79	-	-	-	-	-	-	-	97
September 2019	-	75	-	-	-	-	80	-	85
October 2019	98	-	-	72	-	69	-	-	-
November 2019	56	53	54	78	84	51	57	84	103
December 2019	48	51	78	75	83	26	68	46	87
January 2020	43	39	52	-	73	44	69	47	82
February 2020	-	-	-	79	-	25	57	39	-
April 2020	-	35	-	72	-	-	53	-	46
May 2020	44	39	48	61	41	33	72	35	48
May 2020	72	51	80	-	-	41	-	72	75
June 2020	30	-	39	45	61	44	-	-	-
Non-smoking pedestrians, persons per hour									
July 2019	330	-	567	-	233	745	1050	-	-
August 2019	295	-	-	-	-	-	-	-	301
September 2019	-	202	-	-	-	-	1025	-	293
October 2019	256	-	-	112	-	387	-	-	-
November 2019	797	206	297	170	698	373	817	357	284
December 2019	354	186	331	85	999	464	921	657	261
January 2020	146	189	187	-	879	580	890	378	238
February 2020	-	-	-	65	-	647	796	179	-
April 2020	-	122	-	133	-	-	528	-	223
May 2020	252	175	248	143	574	286	1005	230	283
May 2020	288	119	270	-	-	567	-	303	214
June 2020	91	-	226	210	507	453	-	-	-

Figure S1. Mask carrying of smokers and mask wearing of non-smoking pedestrians during since-outbreak period

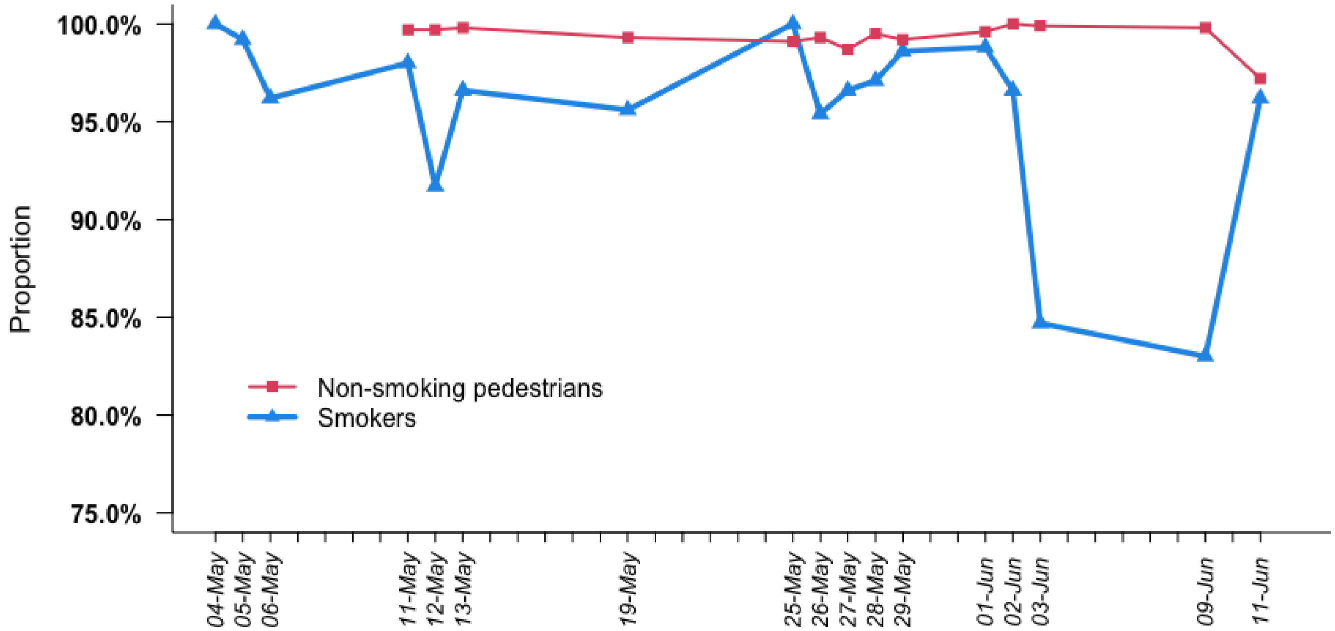

Supplement: Supplementary file 1 [file ijerph-18-01031-s001.pdf]
